# MtDNA mutations and aging—not a closed case after all?

**DOI:** 10.1038/s41392-021-00479-6

**Published:** 2021-02-10

**Authors:** Alexander M. Wolf

**Affiliations:** Laboratory for Morphological and Biomolecular Imaging, Faculty of Medicine, Nippon Medical School, Tokyo, Japan

**Keywords:** Senescence, Cancer metabolism, Ageing, Genomic instability

In a recent paper in *Nature Cancer*, Smith et al. demonstrate that mitochondrial DNA (mtDNA) mutations can confer a growth advantage to intestinal tumors by remodeling cell metabolism, particularly toward elevated de novo serine synthesis.^1^ For the reasons outlined below, in the past decade, the field of mtDNA biology has experienced a relative loss of relevance. The findings of Smith et al. breathe welcome new life into the complex field of mtDNA contributions to aging and cancer.

Compared to nuclear DNA (nDNA), mtDNA experiences high rates of mutation because it is relatively unprotected, mtDNA repair mechanisms are far less sophisticated, and mtDNA is located close to a major source of reactive oxygen species (ROS), the mitochondrial electron transport chain. MtDNA mutations accumulate with age, and it was long thought that they might contribute to the functional decline seen in aging. This idea was dealt a lethal blow when a mouse accumulating mtDNA mutations at vastly increased rates did not show accelerated aging.^2^ This decisive elimination of mtDNA from the pantheon of grand aging theories seemed to leave no important role for accumulating mtDNA mutations in aging.

The dynamics of normal and mutant mtDNA are complex not only in intestinal carcinogenesis. Cells usually contain hundreds of mtDNA molecules, each replicating according to metabolic demand and independent of cell division. Damaged mtDNA molecules need to accumulate and replace most normal mtDNA within a cell to cause mitochondrial efficiency to drop significantly. Why this accumulation happens nonetheless is still poorly understood, but might be driven in part by faster replication of truncated mtDNA and increased mtDNA replication frequency induced by the energy crisis. On the level of cells, however, it is clear that in the aging human intestine, cells harboring large amounts of mtDNA mutations gradually expand and form patches of respiration-deficient cells. This clonal expansion of respiration-deficient cells is, however, not observed in the mouse intestine, and the lack of a good murine model is still hampering research. Smith et al. combine mice homozygous for a proofreading-deficient polymerase γ (which accumulate very high numbers of mtDNA mutations) with a model of intestinal tumorigenesis, tamoxifen-inducible deletion of adenomatous polyposis coli in intestinal crypt stem cells. The difficulty of replicating the human situation of expanding clones of respiration-deficient cells is illustrated by the fact that they still resort to using homozygous polymerase γ mutant mice, even though heterozygous mice also display expanding clones but no premature aging phenotype.

Smith et al. nicely show that while the number of tumors was unaffected by the presence of mtDNA mutations, mtDNA mutations increased tumor growth by inducing metabolic remodeling towards anabolic growth. This hints at positive selection for mtDNA mutation-containing cells and is supported by recently reported enrichment of mtDNA mutations not only in colorectal but also in several other, notably kidney, cancer types.^3^ The finding of altered one-carbon metabolism following respiratory chain disturbances is not new.^4^ Crucially, the same metabolic rewiring is also observed in many types of cancer, where it is critical for growth and cell survival in the nutrient-starved environment of a growing cancer.

It is my personal opinion that most of the many phenotypes that constitute aging are nothing more than a consequence of tumor-suppressive mechanisms that had to evolve to limit the danger posed by expanding clones of cells in large, long-lived organisms like us. The tumor-suppressive mechanism is cellular senescence and irreversible growth arrest. Smith et al. provide welcome new evidence that, in this view, not only oncogenic nuclear DNA mutations contribute to cancer and aging, but, indirectly, also mtDNA mutations. In this view, illustrated in Fig. 1, the clonal expansion of cells and their tumorigenic potential, but not loss of any function or cell death, is, for the organism as a whole, the only relevant consequence of somatic mutations in nDNA and the mechanistic driver of aging. The beautiful results of Smith et al. show that the same can be true for mtDNA mutations. At least in some organs, mtDNA mutations can function as an oncogenic mutation by inducing metabolic remodeling, a bottleneck in oncogenic transformation. In other organs, mtDNA mutations might have the opposite effect, though.^3^

**Fig. 1:**
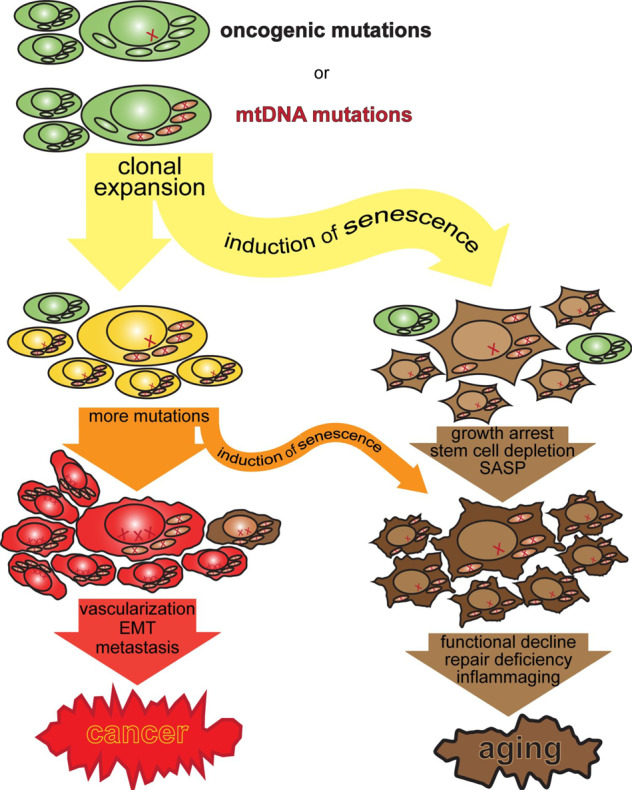
Cancer and aging as the dual consequences of somatic nDNA and mtDNA mutations. Oncogenic and mtDNA mutations drive clonal expansion, which triggers tumor-suppressive cell senescence but eventually leads to the characteristic loss of repair and regenerative capacity in aging. What we experience as aging is simply, and mostly, the consequence of a tumor-suppressive irreversible growth arrest that has evolved to limit the tumorigenic potential of clonally expanding cells

So mtDNA mutations, by contributing to oncogenesis contribute to aging after all, but only by driving oncogenesis. Cellular senescence suppresses cancer but causes the aging phenotype (Fig. 1). A commentary on the results of ref. ^2^ was titled “Mitochondrial DNA mutations and aging: a case closed?” and stated that “The conclusion seems inescapable that mtDNA point mutations are not causally related to the aging process, at least in mice”. While this probably remains true for a direct contribution towards any of the functional decline seen in aging, Smith et al.^1^ and Yuan et al.^3^ firmly establish mtDNA contributions to tumorigenesis. Smith et al. also nicely show that mtDNA mutations accelerate cell proliferation and reduce apoptosis,^1^ increasing the organismal burden of senescent cells. They also show that clonally expanding but anatomically normal human colonic crypts upregulate serine synthesis in response to mitochondrial deficiency.^1^ This shows that mtDNA mutation-driven metabolic rewiring drives clonal expansion, and therefore cellular senescence and aging, even in the absence of overt tumorigenesis. So, after all, mtDNA mutations can still contribute to what we experience as aging.

## Note added to proof

The authors start the introduction with the unfortunate statement “Fundamental changes in the cellular metabolism of tumor cells were first observed in 1956 by Otto Warburg …”.^1^ Warburg observed and reported this more than 30 years earlier in several publications, starting in 1924 with “Über den Stoffwechsel der Carcinomzelle”.^5^
